# Localization of AML-related nucleophosmin mutant depends on its subtype and is highly affected by its interaction with wild-type NPM

**DOI:** 10.1371/journal.pone.0175175

**Published:** 2017-04-06

**Authors:** Barbora Brodská, Markéta Kráčmarová, Aleš Holoubek, Kateřina Kuželová

**Affiliations:** Institute of Hematology and Blood Transfusion, Prague 2, Czech Republic; University of Kansas Medical Center, UNITED STATES

## Abstract

Mutations of the gene for nucleophosmin (NPM1) are the most frequent genetic aberration in patients with acute myeloid leukemia (AML). The mechanism of leukemic transformation in this leukemia subtype is not fully understood, but aberrant cytoplasmic localization of mutated NPM (NPMmut) is widely considered as an important factor for leukemia manifestation. We analyzed the subcellular localization of three types of NPM with a C-terminal mutation (A, B and E). Genes for the individual NPM forms were fused with a gene for one of fluorescent protein variants in plasmids, which were transfected into three cell lines with different endogenous NPM expression. Subcellular localization of the fluorescent protein-labeled NPM was further correlated with the relative expression of all NPM forms. We confirmed a high cytoplasmic expression of NPMmutA and NPMmutB whereas a substantial fraction of NPMmutE was found to be localized in nucleoli. Moreover, we revealed that the localization of fluorescently labeled NPM is affected by the interaction between various forms of the protein.

## Introduction

The phosphoprotein nucleophosmin (NPM) is an abundant protein located mainly in the nucleolus, although it shuttles between the nucleolus, the nucleus and the cytoplasm. It regulates many cellular processes, mainly the ribogenesis [1], centrosome duplication control [2] and apoptosis [3,4]. Mutation of the *NPM1* gene is the most frequent genetic aberration in AML and generally causes NPM relocation from the nucleolus into the cytoplasm [5]. The mistargeting is caused by mutations in exon 12 of the *NPM1* gene leading to the loss of tryptophan W288 and/or W290 at the C-terminus of the resulting protein [6]. The mutations highly compromises the nucleolar localization signal (NoLS) and, moreover, the protein acquires an extra nuclear export signal (NES) in addition to two NESes already present in its N-terminal domain [7]. Specific NPM mutations are characteristic for about 60% of adult AML with the normal karyotype [8] and are associated with good response to induction therapy [9]. The most frequent AML-related NPM mutation type (type A) occurs in 75–80% of adult AML patients with NPM mutation [5,9–11]. The resulting mutated protein (NPMmutA) lacks both tryptophans W288 and W290 and it has the most frequent NES motif L-xxx-V-xx-V-x-L [12]. Bolli et al [13] identified six different NES motifs with various exporting efficiency associated with C-terminal mutations. The strongest NES motifs were associated with NPM mutants retaining W288 that drives the NPM into the nucleolus. The authors concluded that a strong NES motif balancing the force of W288 allows for the export of NPMmut into the cytoplasm, and that NPM translocation might be critical for leukemogenesis. All AML-related NPM mutations reported to date were heterozygous, i.e. the patients were heterozygous for the mutation and retained a wild-type allele [5,9,10]. Homozygous Npm1 mutant knock-in mice were reported to show embryonic lethality [14].

The impact of the mutation type on survival characteristics was widely examined and the results of individual studies varied: while no difference in the overall survival (OS) and in the disease-free survival (DFS) was observed by Pastore et al. [15], other researchers reported either better or worse outcome for patients with NPMmutA vs. patients with mutations of other type [16,17]. The role of different types of *NPM1* mutation, either individually or in the presence of other common gene mutations was suggested to be essential also for childhood AML prognosis [18]. However, these studies generally compared the group of patients with the most frequent mutational type A versus a merged group of patients with other types of mutation. We believe that if the cytoplasmic localization of NPM is critical for leukemogenesis, the difference should be searched between types causing different subcellular localization. We thus compared the subcellular distribution of NPMmutA with that of NPMmut type B, which differs from the type A only in one aminoacid (L289M) and with the type E, which retains W288 and has the strongest NES motif, L-xxx-L-xx-V-x-L [19].

NPM conformation exhibits monomer–pentamer equilibrium, which is modulated by posttranslational modifications, in particular by phosphorylation, and by protein binding [20], the pentamers being formed through the domain located at the N-terminus of the protein [21]. This domain is also responsible for the majority of interactions of NPM with various proteins [1,22]. It was reported, that the ability to oligomerize is, at least in part, maintained in C-terminal mutants [23]. Falini et al [24] suggested that the increased nucleophosmin export into the cytoplasm probably perturbs multiple cellular pathways by loss-of-function (delocalization of NPM nucleolar interactors into the cytoplasm) and/or gain-of-function mechanisms. Balusu et al [25] demonstrated that AML cells expressing mutated NPM are more sensitive to disruptive effects of the inhibitor NSC348884 on NPM oligomerization, in comparison with AML cells expressing NPMwt. Recently, we revealed that the localization of NPMmutA is not exclusively cytoplasmic and that a substantial fraction of NPMmutA still resides in the nucleoli [26]. Moreover, we and other authors [26,27] have shown that due to heterooligomer formation, subcellular distribution of NPMmutA changes when the cells are co-transfected with NPMwt. In the present work, we used HEK-293T cell system allowing high amplification of transfected plasmids to investigate the localization of various mutation types. The impact of the endogenous NPM was then analyzed in three cell lines with different ratio of endogenous to exogenous NPM expression. The interaction between various NPM types was further confirmed by co-immunoprecipitation.

## Material and methods

This study was conducted in the period 02-11/2016.

### Cell culture and chemicals

Cancer cell lines HEK293T (gift from Dr. Š. Němečková, Institute of Hematology and Blood Transfusion, Czech Republic) and NIH 3T3 (gift from Dr. M. Jiroušková, IMG CAS, Czech Republic) were cultivated in DMEM (Sigma-Aldrich), 10% FCS, 37°C and 5% CO_2_ atmosphere. Cancer cell line HeLa (gift from Dr. J. Malínský, IEM CAS, Czech Republic) was cultivated in RPMI 1640 (Biochrom AG) supplemented with 10% FCS, 37°C and 5% CO_2_ atmosphere. Peripheral blood mononuclear cells (PBMC) of AML patients were isolated from leukapheretic products using density gradient centrifugation on Histopaque 1077 (Sigma-Aldrich Corporation, USA) at 500 g and 20°C for 25 min. PBMC were resuspended at a density of 5x10^6^ cells/ml in RPMI 1640 medium (10% FCS, 37°C, 5% CO_2_). All patients signed informed consent to the use of their biological material for research purposes in agreement with the Declaration of Helsinki. The Ethics Committee of the Institute of Hematology and Blood Transfusion approved this research at the application of grant No 16-30268A. All samples were tested for presence of C-terminal NPM mutation by PCR and the mutation type was determined by sequencing as described previously [28].

### Plasmid construction and cell transfection

As described in detail previously [26], gene for nucleophosmin was amplified from cDNA library (Jurkat cells, Origene) by PCR and inserted to vectors peGFP-C2 and pmRFP1-C2 (originally Clontech) designed for expression of protein chimeras with a fluorescent protein connected to the N-terminus of the target protein by standard methods of molecular cloning. NPM mutants were constructed by PCR using extended primers containing mutated part of exon 12 of the *NPM1* gene and restriction sites (Table 1). After amplification in E. coli, the plasmids with subcloned genes were purified with PureYield Plasmid Miniprep System (Promega) and transfected into adherent cell lines using jetPRIME transfection reagent (Polyplus Transfection) for each experiment. Transfection efficiency was analyzed by flow cytometry (BD Fortessa).

**Table 1:** Sequence of extended primers used for construction of the NPM mutants.

Mutation type	Forward primer	Reverse primer
NPM mutA	AAAAAACTCGAGCATGGAAGATTCGATGGACATAG	AATTAAGGATCCACTATTTTCTTAAAGAGACTTCCTCCACTGCCAGACAGAGATCTTGAATAGCCTCTTGGTCAG
NPM mutB	AAAAAACTCGAGCATGGAAGATTCGATGGACATAG	AATTAAGGATCCACTATTTTCTTAAAGAGACTTCCTCCACTGCCATGCAGAGATCTTGAATAGCCTCTTGGTCAG
NPM mutE	AAAAAACTCGAGCATGGAAGATTCGATGGACATAG	AATTAAGGATCCACTATTTTCTTAAAGAGACTTGGGCAAGAGACTGCCAGAGATCTTGAATAGCCTCTTGGTCAG

### Immunofluorescence

The samples were prepared as described previously [26]. Briefly, cells in suspension were seeded on a coverslip in humidified chamber for 15 min and then fixed with 4% paraformaldehyde (PFA) overnight at 4°C. After 10min of permeabilization by 0,5% Triton X-100, the cells were incubated for 1h with a mouse monoclonal anti-NPM primary antibody (clone 3F291, Santa Cruz Biotechnology, 1:100) and for another 1h with the secondary antibody (AlexaFluor555-conjugated anti-mouse, Life Technologies, 1:200) and with Hoechst33342 (1μM, Life Technologies). The stained cells were observed under confocal laser scanning microscope FluoView FV1000 (Olympus Corporation).

#### Live-cell imaging

Subcellular distribution and colocalization of eGFP- or mRFP1-fused variants of nucleophosmin was observed by Olympus FluoView FV1000 confocal microscope (Olympus Corporation). For subcellular distribution statistics, at least 800 cells from three independent experiments were evaluated. Fluorescence images were processed by FluoView software FV10-ASW 3.1.

### Cell lysis

Transfected adherent cells were briefly washed with PBS, trypsinized and extensively washed with PBS. The cell pellets were lysed in Laemmli sample buffer, boiled for 5 min, centrifuged at 200.000g/4°C for 4h and the supernatant was stored at -20°C.

### Immunoprecipitation

GFP-Trap_A system (Chromotek) was used following the manufacturer´s instructions. Briefly, transfected adherent cells were resuspended in ice-cold PBS, scrapped from dish and extensively washed with PBS. Then the cell pellet was lysed in the lysis buffer (10mM Tris/Cl pH7.5, 150mM NaCl, 0.5mM EDTA, 0.5% NP-40, protease and phosphatase inhibitors), incubated on ice for 30 min and centrifuged at 20.000g/10min/4°C. The lysate was then transferred into the GFP-Trap_A beads and incubated for 1h at 4°C. After centrifugation and extensive wash in the diluting buffer (10mM Tris/Cl pH7.5, 150mM NaCl, 0.5mM EDTA), GFP-Trap_A beads were resuspended in SDS-sample buffer, boiled for 5 min and centrifuged at 2.500g/2min/4°C. Supernatant was stored at -20°C until used for SDS-PAGE.

### Western blotting

Five microliters of each sample were subjected to SDS-PAGE and transferred into nitrocellulose membrane (Hybond PVDF, Amersham). Mouse monoclonal antibodies against β-actin, GFP and NPM (clone NA24 for wt+mut detection, clone E3 for wt-only detection) were from Santa Cruz Biotechnology. All primary antibodies were used at a dilution 1:100–1:500. Anti-mouse HRP-conjugated secondary antibody was purchased from Thermo Scientific and used at concentrations 1:10,000–1:50,000. ECL Plus Western Blotting Detection System (Amersham) was used for chemiluminescence visualization and evaluation by G-box iChemi XT4 digital imaging device (Syngene Europe).

### Statistical analysis

No power calculations were performed. We analyzed all primary AML samples available at the Institute of Hematology and Blood Transfusion during the period 2015–2016 (N = 17). The majority of experiments were performed using cell lines and repeated until the observed differences between groups reached statistical significance. A p-value of 0.05 or lower was pre-set to be indicative of a statistically significant difference between groups compared. In diagrams, arithmetic means of at least three replicates of all experiments were plotted with SD error bars. Significance levels (p values of ANOVA or Student´s t-test) were determined using InStat Software (GraphPad Software).

## Results

### Subcellular localization of mutated NPM depends on mutation type

**Fig 1:**
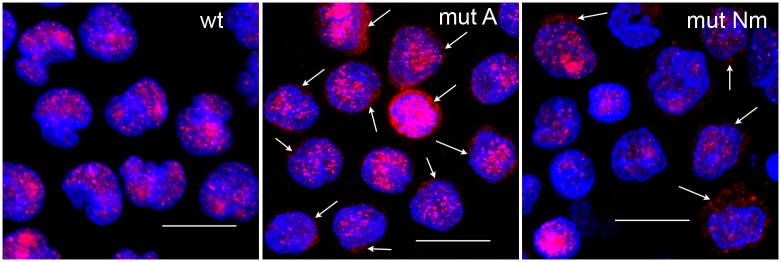
NPM is localized in the cytoplasm of blast from AML patients with NPM mutation. PBMC from AML patients with NPMwt (wt), NPMmutA (mut A) or NPMmutNm (mut Nm) were incubated with anti-NPM (clone 3F291) primary and AlexaFluor555 secondary antibodies (red). The nuclei were visualized with Hoechst 33342 (blue). Arrows indicate the cytoplasmic localization of NPM in AML blasts with NPMmut. The bars represent 10μm.

Seventeen PBMC samples from AML patients were screened for the presence of NPM mutation by the PCR and by the immunofluorescence. We detected the NPMwt in 7 (41%) patients, the NPMmutA in 9 (53%) patients and one patient had the mutation type Nm (1108_1109ins CCAG). Blasts with extranuclear NPM localization were found in all samples from the patients with a NPM mutation whereas the localization of NPM was restricted to nucleoli (and partially nuclei) in the samples without mutation (Fig 1).

**Fig 2:**
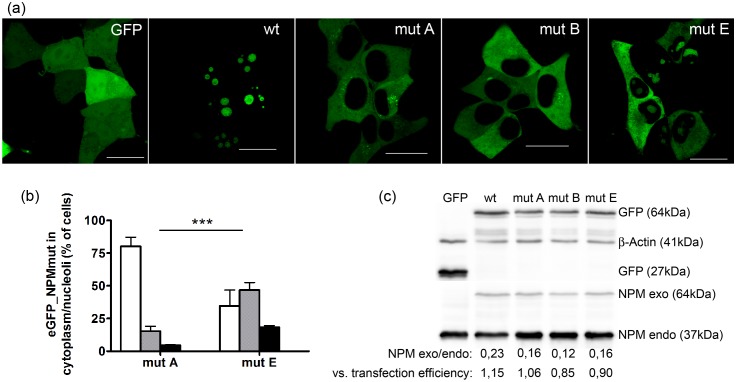
Subcellular distribution of mutated NPM depends on mutation type. (a) eGFP fluorescence from HEK-293T cells transfected with eGFP plasmid (GFP), eGFP_NPMwt (wt), eGFP_NPMmutA (mutA), eGFP_NPMmutB (mutB) or eGFP_NPMmutE (mutE) showing various subcellular distribution of individual NPM variants. The bars represent 20μm. (b) fraction of transfected cells displaying eGFP_NPMmutA (or E) signal only from the cytoplasm (white bars), from the cytoplasm and nucleoli (grey bars) or only from nucleoli (black bars). The error bars in the graph represent ±SD of at least 3 independent experiments. Statistical significance degree of difference between mutA and mutE obtained from two-way ANOVA test was P < 0.001 (***). (c) immunoblot of lysates from HEK-293T cells transfected with individual NPM variants. GFP-NPM (exogenous) is detected at 64 kDa, the endogenous NPM at 37 kDa. β-Actin represents the loading control. Densitometric evaluation of NPM exo/endo level and the ratio of NPMexo/endo expression vs the transfection efficiency (20%, 15%, 13,9% resp. 17,8% for wt, mutA, mutB resp. mutE) are indicated for the individual cell lines.

We transfected HEK-293T cell line with eGFP-labeled variants of NPM and examined the eGFP_NPM subcellular localization under the confocal microscope (Fig 2a). The wild-type NPM and three types of mutated NPM (A, B and E) were analyzed. While the eGFP_NPMwt was detected solely in nucleoli, more than 80% of eGFP_NPMmutA-transfected cells exhibited exclusively cytoplasmic localization of the mutated protein (Fig 2b). A combination of eGFP_NPMmutA signal from the nucleolus with cytoplasmic staining was observed in approximately 15% of the transfected cells. Moreover, the relative fluorescence intensity from the remaining cells, showing eGFP_NPMmutA signal only in the nucleoli (approximately 5% of transfected cells), was weak, indicating low plasmid amplification in these cells. Subcellular distribution of eGFP signal in cells transfected with eGFP_NPMmutB was almost identical as for NPMmutA (Fig 2a). On the contrary, 65% of cells transfected with eGFP_NPMmutE displayed eGFP fluorescence from the nucleolus, whether exclusively or partially (i.e. signal was detected from both the cytoplasm and the nucleoli) (Fig 2a and 2b, S1 Table). Identical results were obtained with plasmids containing the red form of the fluorescence protein, mRFP1, instead of eGFP (data not shown). The transfection efficiency measured by flow cytometry was about 45% in all samples and high expression of recombinant fusion proteins was confirmed by immunoblot (Fig 2c, S1 Fig).

### Interaction between wild-type and mutated NPM

**Fig 3:**
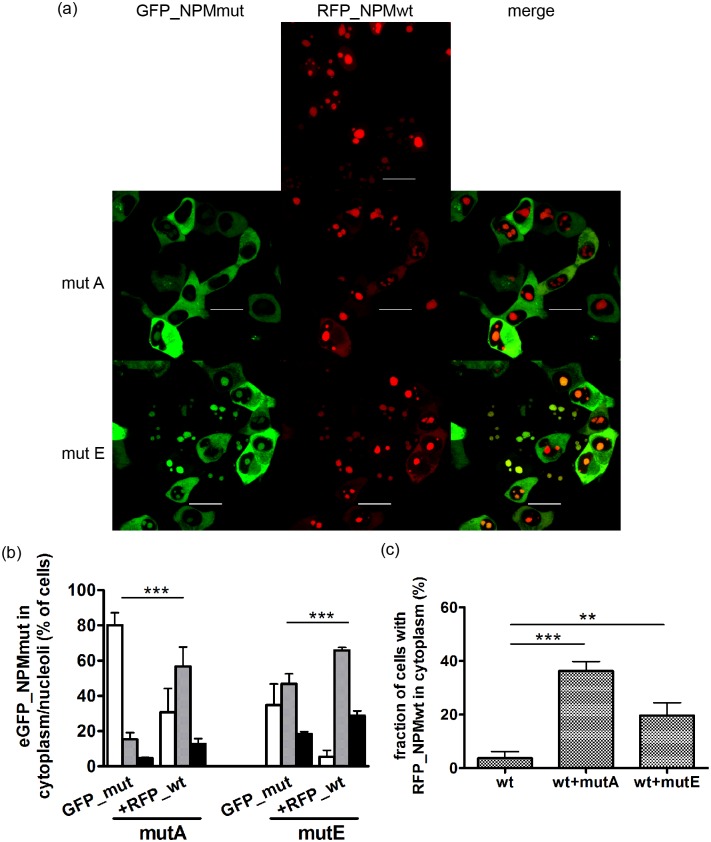
Interaction between wild-type and mutant affects localization of individual forms of NPM. (a) eGFP (green) and mRFP1 (red) fluorescence from HEK-293T cells co-transfected with mRFP1_NPMwt and eGFP_NPMmutA (mutA) or eGFP_NPMmutE (mutE). The bars represent 20 μm. (b) fraction of transfected cells displaying eGFP_NPM signal only from the cytoplasm (white bars), from the cytoplasm and nucleoli (grey bars) or only from nucleoli (black bar). GFP_mut denotes the signal from cells transfected with eGFP_NPMmut only, +RFP_wt denotes eGFP signal from cells co-transfected with eGFP_NPMmut and mRFP1_NPMwt. The error bars in the graph represent ±SD of at least 3 independent experiments. (c) fraction of transfected cells displaying mRFP1_NPMwt signal from the cytoplasm: wt—cells transfected only with RFP_NPMwt, wt+mutA (or E)–cells co-transfected with RFP_NPMwt and GFP_NPMmutA (or E). The error bars in the graph represent ±SD of 5 independent experiments. Statistical significance degree of difference between the samples: P < 0.01 (**), P < 0.001 (***).

We have previously checked the tug-of-war hypothesis described by Bolli et al [27] suggesting that the localization of both fluorescently labeled wt and mutated NPM forms depends on their mutual ratio. We showed that the abundance of one NPM form caused partial redistribution of its oligomer partner in Hela cells co-transfected with eGFP_NPMmutA and mRFP1_NPMwt [26]. Here we analyzed the distribution of fluorescently labeled NPM variants in HEK-293T cells co-transfected with mRFP1_NPMwt and eGFP_NPMmutA or eGFP_NPMmutE (Fig 3a). For both mutation types, co-transfection with wt caused significant changes in the subcellular distribution: higher fraction of eGFP_NPMmut in the nucleolus as well as a fraction of mRFP1_NPMwt in the cytoplasm was observed in comparison with the distribution in single form-transfected cells (Fig 3b and 3c, S1 and S2 Tables). Our observations prove the fact that the ability of NPM to form oligomers is not disrupted by any type of C-terminal mutation and that heterooligomers between the wild-type and mutated NPM are formed affecting the localization of each other.

### Endogenous NPM affects the localization of NPMmut

**Fig 4:**
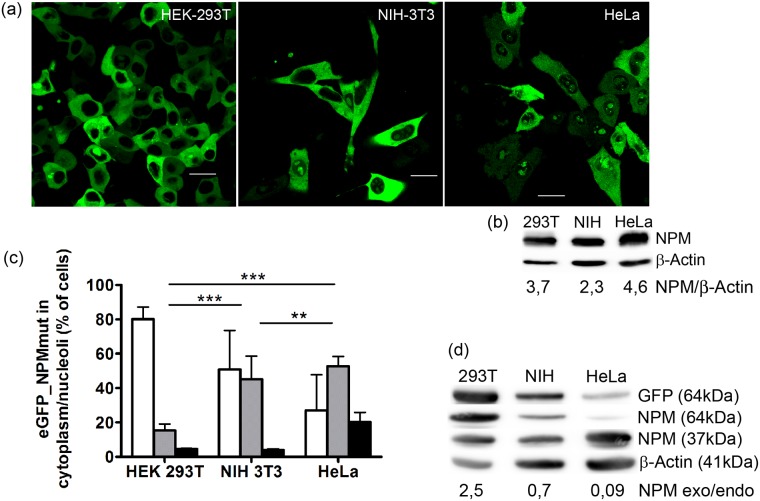
Localization of exogenous NPMmutA depends on endogenous NPM level. (a) eGFP fluorescence from HEK-293T (1), NIH-3T3 (2) or HeLa (3) cells transfected with eGFP_NPMmutA showing its various subcellular distribution in individual cell lines. The bars represent 20μm. (b) immunoblot of lysates from various cell lines indicates different endogenous NPM expression. β-Actin represents the loading control. Densitometric evaluation of NPM/β-Actin ratio is indicated for individual cell lines. (c) fraction of transfected cells displaying eGFP_NPM signal only from the cytoplasm (white bars), from the cytoplasm and nucleoli (grey bars) or only from nucleoli (black bar). The error bars in the graph represent ±SD of at least 3 independent experiments. Statistical significance degree of difference between the samples: P < 0.01 (**), P < 0.001 (***). (d) immunoblot of lysates from various cell lines transfected with NPMmutA indicates different expression of transfected eGFP_NPM. GFP-NPM (exogenous) is detected at 64 kDa, the endogenous NPM at 37 kDa. β-Actin represents the loading control. Relative ratio of NPM exo/endo expression is indicated for the individual cell lines. Two-fold concentrations of primary and secondary antibodies had to be used to detect exogenous NPM expression in all lines. Therefore, absolute evaluation of the NPM exo/endo expression needs correction for the exo/endo NPM ratio calculated in Fig 2c.

The subcellular distributions of NPMmutA in both single (NPMmut only) or double (NPMmut+NPMwt) transfected HEK-293T cells markedly differed from the distribution previously observed in HeLa cells [26]. We hypothesize, that the reason for this difference lays in various endogenous expression of NPM in these two cell lines. Karyotype studies of HeLa cells proved a multiplied number of NPM gene copies [29] and a high endogenous NPM expression was thus expected in this cell line. On the other hand, HEK-293T cell line contains the SV40 Large T-antigen, which allows for amplified expression from transfected plasmids containing the SV40 origin of replication. Therefore, we compared the localization and level of NPM protein expression in these two cell lines. In addition, the commonly used mouse NIH-3T3 cell line with standard endogenous NPM expression and unaffected plasmid amplification was analyzed for comparison (Fig 4, S2 Fig, S3 Table). The distribution of eGFP-NPMmutA varied from almost cytoplasmic in HEK-293T to highly nucleolar in HeLa (Fig 4a and 4c, S3 Table). The expression of the endogenous NPM was higher in HeLa compared to the other cell lines (Fig 4b, S2 Fig) and the ratio between the endogenous and the exogenous protein in the transfected cells (Fig 4d, S2 Fig) reflected the high amplification ability of HEK-293T (even with correction for various transfection efficiency in individual cell lines, Table 2).

**Table 2:** Transfection efficiency for individual cell lines assessed by flow-cytometry.

	HEK-293T	NIH 3T3	HeLa
**transfection efficiency (% of cells)**	47 ± 13	11 ± 4	19 ± 4
**estimated ratio NPM endo:NPM_GFP**	1: 1	4: 1	10: 1

mean±SD values from at least 6 samples were calculated. Ratio of NPM forms was estimated from the transfection efficiency and the protein expression levels determined from WB.

**Fig 5:**
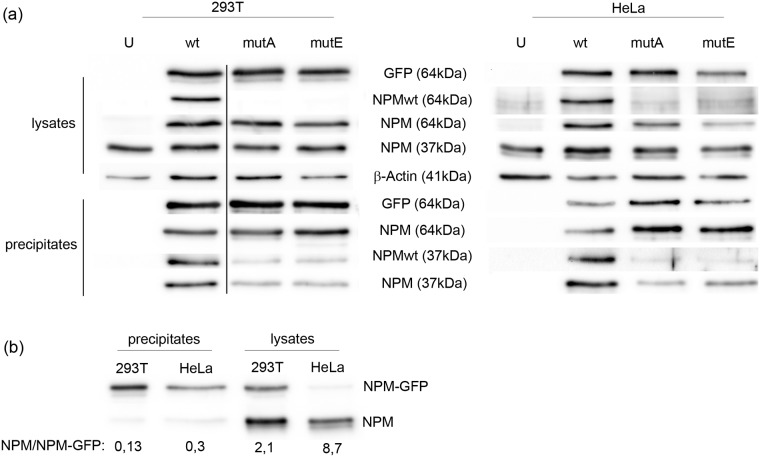
Formation of heterooligomers between eGFP_NPM and endogenous NPM was confirmed by GFP-precipitation. (a) Representative immunoblots of lysates and GFP-precipitates from the cells transfected with individual NPM variants. U: untransfected cells, wt: eGFP_NPMwt, mutA: eGFP_NPMmutA, mutE: eGFP_NPMmutE. Anti-NPM antibody clone NA24 was used to detect the overall NPM expression (i.e. both the NPMwt and NPMmut), the clone E3 was used to detect NPMwt only. GFP-NPM (exogenous) is detected at 64 kDa, the endogenous NPM at 37 kDa. β-Actin represents the loading control. (b) The ratio between endogenous (NPM) and exogenous (NPM-GFP) expression in GFP-precipitates and lysates from cells transfected with eGFP_NPMwt. The membrane from 30 to 100 kDa was incubated with anti-NPM clone NA24.

A good correlation between the fraction of cells with cytoplasmic-only NPMmutA localization and the ratio of exogenous vs. endogenous NPM expression was observed. We suggest that heterodimers are formed not only between the fluorescently labeled NPM forms but also between the recombinant and the endogenous protein. This suggestion was further confirmed by eGFP-precipitation from lysates of transfected cells of HEK-293T and HeLa cell lines using GFP-Trap nanobeads (Fig 5a, S3 Fig). NPM expression was examined by two anti-NPM antibodies. The anti-NPM clone NA24 is directed to recognize the N-terminus of the human NPM and it is thus able to detect the overall NPM, i.e. both the NPMwt and NPMmut. The anti-NPM clone E3 is specific for an epitope at the C-terminus (aa 253–294) of NPMwt and it should hardly recognize the NPMmut. Indeed, whereas the clone NA24 detected GFP-NPM signal from all lysates of transfected cells, the clone E3 generated a signal only from samples transfected with eGFP-NPMwt. On the contrary, both clones equally detected the endogenous NPM in all precipitates containing any type of eGFP_NPM but not in precipitates from untransfected cells. Despite its relatively low expression in HeLa cells, eGFP_NPM effectively co-precipitated the endogenous NPM also in this cell line. Moreover, a higher ratio of the co-precipitated NPMwt vs. the precipitated eGFP_NPM corresponds to higher expression of the endogenous NPM in HeLa cells (Fig 5b, S3 Fig). For both cell lines, the amount of co-precipitated NPM was substantially higher in the samples from eGFP_NPMwt-transfected cells than in the samples transfected with eGFP_NPMmut.

## Discussion

The significance of specific nucleophosmin mutations in AML has been recognised by the World Health Organization (WHO) which defined the AML with NPM1 mutation as a distinct entity [24]. The AML with NPM1 mutation without concomitant mutations in other genes is classified into the group with favorable prognosis. However, leukemogenic potential of the mutation as well as the reason for the better outcome are still unclear. The most frequent mutation type (type A) occurs in 75% of patients with NPM mutation, other relatively frequent types B, resp. D are detected in about 9, resp 8% of patients [10,15,30–32]. The mutations A and D differ from each other only in one base of the inserted tetranucleotide, without change in the resulting aminoacid sequence. The type B differs from these types also in a single base, which results in the change in one aminoacid (L289M) in the translated protein [33]. All the proteins resulting from the most frequent mutation types lack both tryptophans W288 and W290 and possess a weak acquired NES motif L-xxx-V-xx-V-x-L. The presence of a NPM mutation in patients with AML was reported to correlate with the cytoplasmic localization of NPM [34]. Consistently with these reports, we observed cytoplasmic NPM localization in all AML samples with NPM mutation (Fig 1). However, the only non-A case in our cohort was of type Nm which is very similar to the type A and the resulting protein differs from NPMmutA in one aminoacid only (L289Q).

The studies investigating the impact of mutation type mostly compared groups of patients with mutation type A versus non-A types. Whereas Koh et al observed worse OS and shorter remission for the non-A group [17], Alpermann et al. reported better survival in patients with non-A mutations [16]. Pastore 2014 [15] found no difference between type A and non-A groups and, moreover, these authors did not find any difference even in a more detailed discrimination between the types A, B, D and the others (rare). Recently, Alpermann et al. [30] reported that different subtypes of *NPM1* mutation were associated with different profiles with respect to clinical parameters as well as to accompanying molecular markers. Particularly, they revealed that *DNMT3A* mutations worsen the outcome of patients with type A and type D *NPM1* mutations but not with the type B. However, statistics matching the mutations according to their putative subcellular distribution were not performed, probably due to the low frequency of the rare mutations. We suggested previously, that the cytoplasmic localization of NPM is critical for immune therapy prognosis [28]. Therefore, it is important to investigate the difference between the mutation types causing different subcellular localization. In the present work, we examined the localization of three types of NPM mutation (A, B and E) in HEK-293T cell line, in relation to the presence of W288 and the force of the acquired NES motif. We uncovered substantial difference between the localization of the NPMmutA (or B) and the NPMmutE. A high proportion of NPMmutE is retained in the nucleoli in contrast to the mostly cytoplasmic localization of NPMmutA (Fig 2). Interestingly, the mean fluorescence intensity (MFI) determined by flow-cytometry as well as the GFP-NPM level analyzed by immunoblot revealed that the amplification of the plasmids was mostly lower in cells transfected with NPMmut than in NPMwt-transfected cells indicating lower amplification of plasmids containing NPMmut.

In agreement with the experiments described by Bolli et al [27], the localization of each NPMmut type was strongly affected by the co-expression of NPMwt, probably due to hetero-oligomer formation. In cells co-transfected with GFP-NPMmut and RFP-NPMwt, a higher proportion of NPMmut in the nucleoli as well as the NPMwt in the cytoplasm was detected for the both A and E mutation types (Fig 3). The interaction between various NPM forms was further tested in three different cell lines representing various expression systems and pools of the endogenous NPM. A nice correlation of GFP-NPMmut localization with the ratio of exogenous vs. endogenous NPM expression was observed (Fig 4 and Table 2). The interaction between the endogenous and exogenous NPM was further evidenced thanks to GFP-precipitation (Fig 5a). The formation of NPM oligomers or complexes with its interaction partners mediated by its N-terminal domain is largely documented [22,35,36] and the ability of NPM to form oligomers was reported to be retained also in its variants with an altered C-terminus, whether in the fusion protein NPM-ALK [37] or in the protein with specific mutation [38]. Nonetheless, little is known about the potential of the oligomerization domain of the altered protein. In our experiments, a higher proportion of co-precipitated endogenous NPM according to the lowest ratio of exo-/endogenous NPM expression was detected in HeLa cells (Fig 5b). Irrespectively of the cell line, the amount of co-precipitated endogenous NPM was substantially higher in cells transfected with GFP-NPMwt than in cells transfected with any type of NPMmut. This may be partially explained by a higher accessibility of the endogenous NPM for eGFP-NPMwt due to their identical localization. Similar localization should favor the interaction of the endogenous NPM with NPMmutE rather than with NPMmutA, but we did not observe any difference between the levels of co-precipitated endogenous NPM in samples with mutations A and E. Hence, it is possible that the oligomerization potential of NPMmut is lowered when compared to the interaction potential of the wild-type form. This can be supported by the findings of Balusu et al [25] that the cells with NPMmut are more susceptible to a specific inhibitor of NPM oligomerization than the cells with NPMwt and that the NPMmut tends to form dimers rather than oligomers. Recently, it was uncovered that unbalanced allelic expression of mutant alleles is a relatively common occurrence in multiple myeloma patients [39]. The mutant/wild-type allelic ratio for NPM1 has been suggested to have a prognostic value in AML [40]. In summary, besides the type of the mutation, the oligomerization potential of NPMmut together with the NPMwt/NPMmut ratio considerably affects the subcellular NPM distribution and likely the patient´s outcome.

## Conclusion

Changes in the intracellular localization contribute very likely to leukemogenicity as well as to the survival advantage which are associated with nucleophosmin mutations in acute myeloid leukemia. Hence, it is important to describe in detail the localization of both the wild-type and the mutated protein in cells with every mutation type. The basic location for the wild-type NPM is in the nucleoli, NPM with mutations A or B reside in the cytoplasm, whereas the form E is found in the cytoplasm, in the nucleus and in the nucleoli. Furthemore, the localization of all these forms is affected by their relative amounts thanks to oligomer formation. Finally, the ability of NPMmut to form oligomers seems to be lowered irrespective of the mutation type.

## Supporting information

S1 Fig Original Western blots with molecular size marker for Fig 2c. (TIF)

S2 Fig Original Western blot with molecular size marker for Fig 4b ((a)) and 4d ((b)). (TIF)

S3 Fig Original Western blots with molecular size marker for Fig 5a ((a)) and 5b ((b)). Multiple blots were performed from identical samples in one SDS-PAGE run for individual cell lines ((a)). (TIF)

S1 Table Subcellular distribution of the mutated NPM type A (mutA) and type E (mutE) in HEK-293T cells transfected with the eGFP_NPMmut (columns mutA/E only) or co-transfected with the eGFP_NPMmut and mRFP1_NPMwt (columns mutA/E + wt). The data from three independent experiments are presented as fractions of cells (% of transfected cells) exhibiting eGFP_NPM signal from the cytoplasm only (C), from the cytoplasm and the nucleoli (C+N) or from nucleoli only (N).
(DOCX)

S2 Table Fraction of transfected cells displaying mRFP1_NPMwt signal from the cytoplasm. wt only—cells transfected only with RFP_NPMwt, +mutA (or E)–cells co-transfected with RFP_NPMwt and GFP_NPMmutA (or E).
(DOCX)

S3 Table Subcellular distribution of the mutated NPM type A in HEK-293T, NIH-3T3 or HeLa cells transfected with the GFP_NPMmutA. The data from at least three independent experiments are presented as fractions of cells (% of transfected cells) exhibiting GFP_NPM signal from the cytoplasm only (C), from the cytoplasm and the nucleoli (C+N) or from nucleoli only (N).
(DOCX)

S4 Table Transfection efficiency of individual cell line transfections with fluorescently labeled forms of NPM. (DOCX)
