# Supplementary figures and images for: Localization of AML-related nucleophosmin mutant depends on its subtype and is highly affected by its interaction with wild-type NPM
Source: PLoS One. 2017 Apr 6;12(4):e0175175. doi: 10.1371/journal.pone.0175175 (PMC5383266; doi:10.1371/journal.pone.0175175)

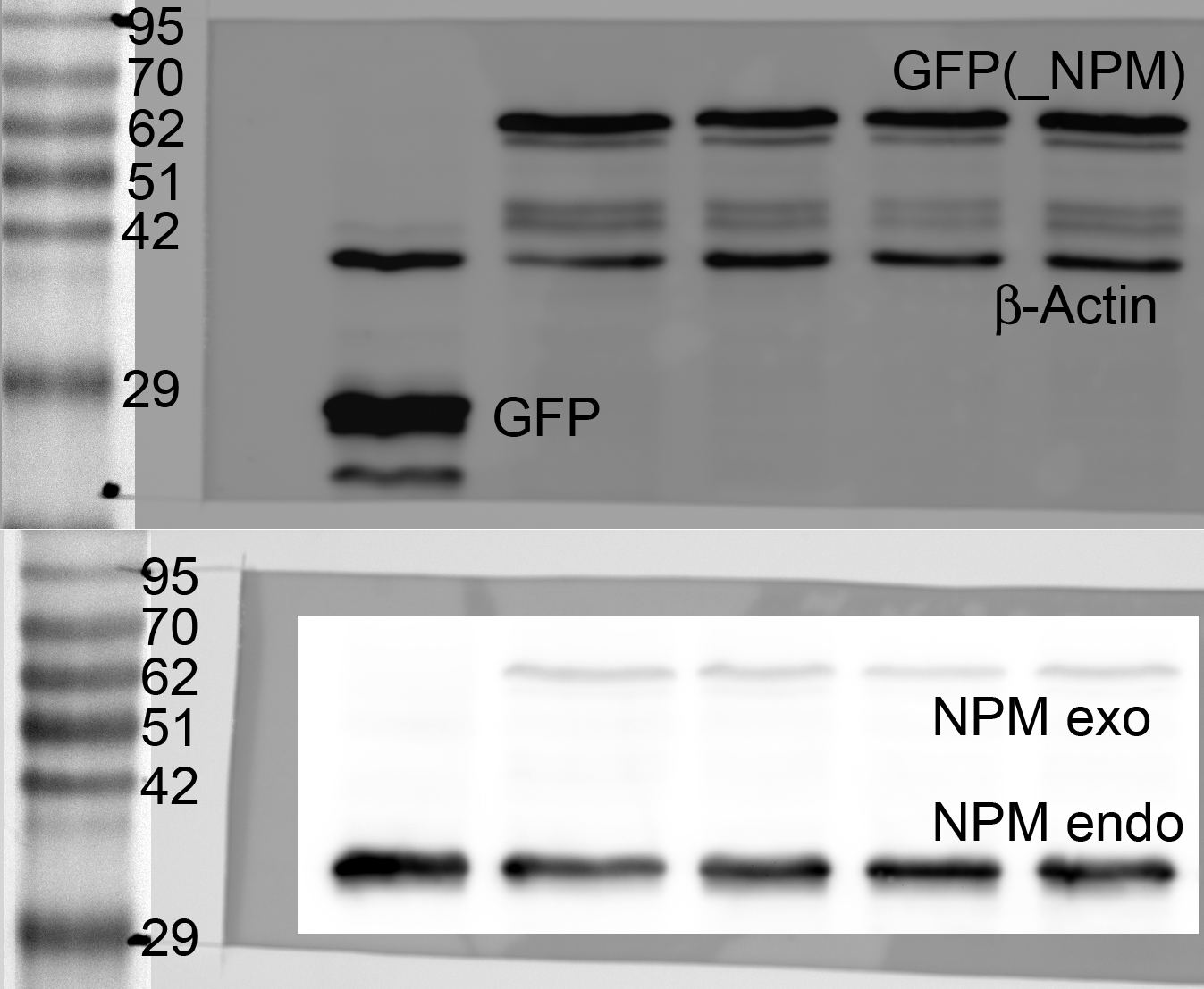

Supplement: S1 Fig — (TIF) [file pone.0175175.s001.tif]

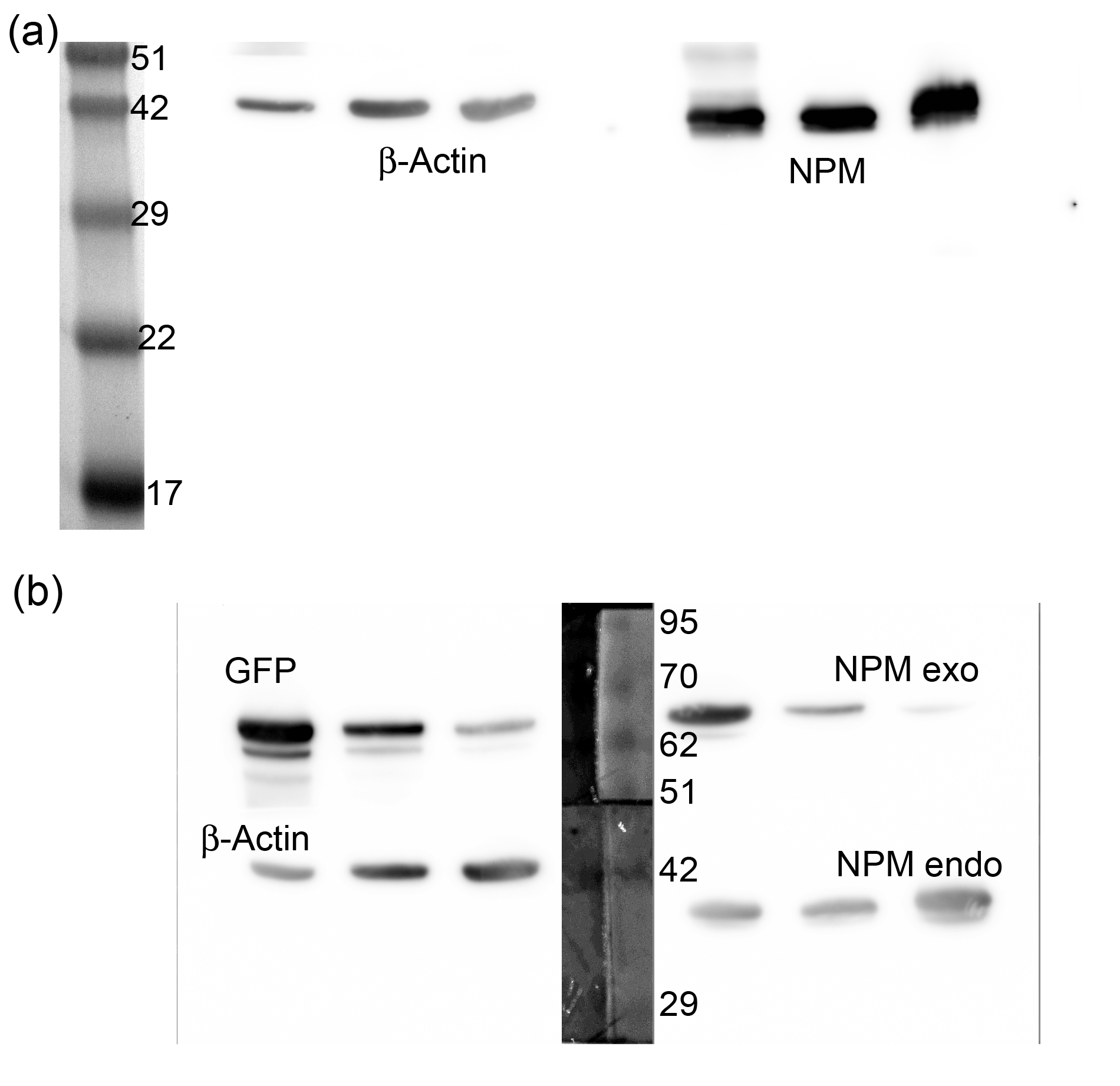

Supplement: S2 Fig — (TIF) [file pone.0175175.s002.tif]

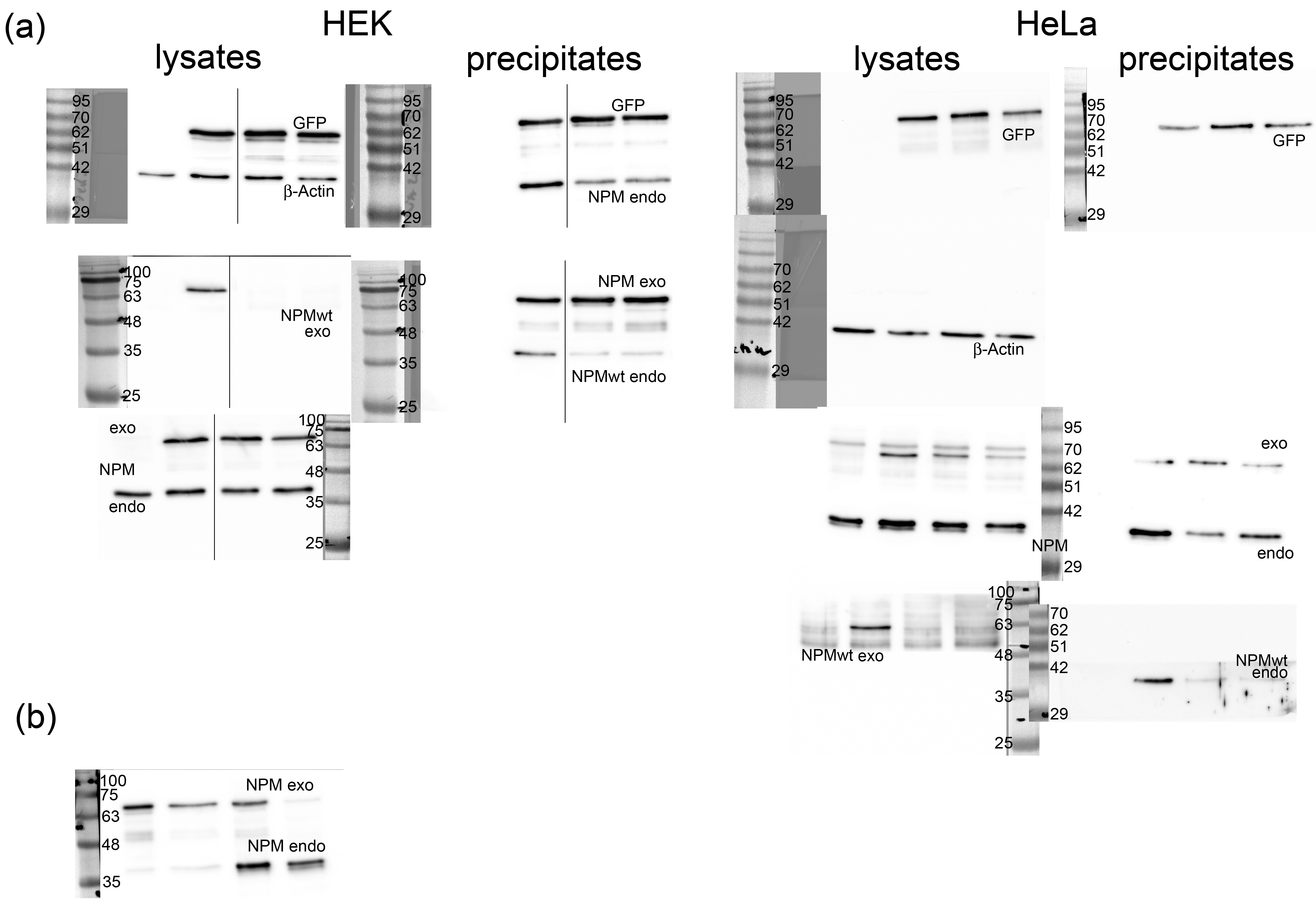

Supplement: S3 Fig — (TIF) [file pone.0175175.s003.tif]
