# Supplementary material for: Localization of AML-related nucleophosmin mutant depends on its subtype and is highly affected by its interaction with wild-type NPM
Source: PLoS One. 2017 Apr 6;12(4):e0175175. doi: 10.1371/journal.pone.0175175 (PMC5383266; doi:10.1371/journal.pone.0175175)
Supplement: S1 Table — The data from three independent experiments are presented as fractions of cells (% of transfected cells) exhibiting eGFP_NPM signal from the cytoplasm only (C), from the cytoplasm and the nucleoli (C+N) or from nucleoli only (N). (DOCX) [file pone.0175175.s004.docx]

| Exper. No. | mutA only | | | mutA + wt | | |
| --- | --- | --- | --- | --- | --- | --- |
|  | C | C+N | N | C | C+N | N |
| 1 | 72 | 23 | 5 | 21 | 70 | 10 |
| 2 | 84 | 12 | 4 | 25 | 65 | 10 |
| 3 | 84 | 11 | 5 | 46 | 35 | 19 |
| Exper. No. | mutE only | | | mutE + wt | | |
|  | C | C+N | N | C | C+N | N |
| 1 | 21 | 58 | 20 | 4 | 69 | 27 |
| 2 | 39 | 43 | 19 | 3 | 63 | 34 |
| 3 | 44 | 40 | 16 | 10 | 65 | 25 |
