# Supplementary material for: Localization of AML-related nucleophosmin mutant depends on its subtype and is highly affected by its interaction with wild-type NPM
Source: PLoS One. 2017 Apr 6;12(4):e0175175. doi: 10.1371/journal.pone.0175175 (PMC5383266; doi:10.1371/journal.pone.0175175)
Supplement: S2 Table — wt only—cells transfected only with RFP_NPMwt, +mutA (or E)–cells co-transfected with RFP_NPMwt and GFP_NPMmutA (or E). (DOCX) [file pone.0175175.s005.docx]

| Exper. No. | % of cells with NPMwt in cytoplasm | | |
| --- | --- | --- | --- |
|  | wt only | +mutA | +mutE |
| 1 | 1 | 39 | 21 |
| 2 | 4 | 40 | 18 |
| 3 | 6 | 32 | 23 |
| 4 |  | 37 | 24 |
| 5 |  | 33 | 12 |
