# Supplementary material for: Localization of AML-related nucleophosmin mutant depends on its subtype and is highly affected by its interaction with wild-type NPM
Source: PLoS One. 2017 Apr 6;12(4):e0175175. doi: 10.1371/journal.pone.0175175 (PMC5383266; doi:10.1371/journal.pone.0175175)
Supplement: S3 Table — The data from at least three independent experiments are presented as fractions of cells (% of transfected cells) exhibiting GFP_NPM signal from the cytoplasm only (C), from the cytoplasm and the nucleoli (C+N) or from nucleoli only (N). (DOCX) [file pone.0175175.s006.docx]

| Exper. No. | HEK 293T | | | NIH 3T3 | | | HeLa | | |
| --- | --- | --- | --- | --- | --- | --- | --- | --- | --- |
|  | C | C+N | N | C | C+N | N | C | C+N | N |
| 1 | 72 | 23 | 5 | 75 | 21 | 4 | 27 | 56 | 17 |
| 2 | 84 | 12 | 4 | 47 | 48 | 5 | 18 | 61 | 21 |
| 3 | 84 | 11 | 5 | 31 | 67 | 2 | 56 | 36 | 8 |
| 4 |  |  |  |  |  |  | 8 | 57 | 35 |
