# Supplementary material for: Localization of AML-related nucleophosmin mutant depends on its subtype and is highly affected by its interaction with wild-type NPM
Source: PLoS One. 2017 Apr 6;12(4):e0175175. doi: 10.1371/journal.pone.0175175 (PMC5383266; doi:10.1371/journal.pone.0175175)
Supplement: S4 Table — (DOCX) [file pone.0175175.s007.docx]

|  | HEK | NIH | HeLa |
| --- | --- | --- | --- |
|  | 61 | 13,2 | 24 |
|  | 41,9 | 12,4 | 20,7 |
|  | 42,5 | 6,4 | 15,6 |
|  | 29,3 | 6,5 | 21,1 |
|  | 56,8 | 11,5 | 19,6 |
|  | 48,7 | 10,4 | 12,1 |
|  | 44,1 | 19 | 15 |
|  | 63,7 |  | 20,4 |
|  | 57,6 |  | 22,2 |
|  | 55,5 |  | 22,2 |
|  | 66,1 |  | 18,5 |
|  | 49,9 |  | 16,8 |
|  | 54,1 |  |  |
|  | 48,2 |  |  |
|  | 38,5 |  |  |
|  | 36,6 |  |  |
|  | 30 |  |  |
|  | 31 |  |  |
|  | 47 |  |  |
|  | 61 |  |  |
|  | 34,8 |  |  |
|  | 33,9 |  |  |
|  | 23,8 |  |  |
|  | 28,4 |  |  |
|  | 20,1 |  |  |
|  | 57,7 |  |  |
|  | 49,8 |  |  |
|  | 66,2 |  |  |
|  | 49,6 |  |  |
|  | 64,9 |  |  |
|  | 43,8 |  |  |
|  | 70 |  |  |
|  | 55 |  |  |
|  | 45 |  |  |
| Mean | 47 | 11 | 19 |
| SD | 13 | 4 | 4 |
